# Quantitative Detection of Pyrene in Edible Oil via Plasmonic TLC-SERS Combined with Machine Learning Analysis

**DOI:** 10.3390/bios15080477

**Published:** 2025-07-23

**Authors:** Jiahui Tian, Xianhe Jiao, Jiaqi Guo, Qian Yu, Shuqin Zhang, Guizhou Gu, Kundan Sivashanmugan, Xianming Kong

**Affiliations:** 1 School of Petrochemical Engineering, Liaoning Petrochemical University, Fushun 113001, China; tianjiahui@stu.lnpu.edu.cn (J.T.); lnpulnpu0413@163.com (X.J.); qyu@lnpu.edu.cn (Q.Y.); xmkong@lnpu.edu.cn (X.K.); 2 Jiangsu Co-Innovation Center for Efficient Processing, Utilization of Forest Resources and Joint International Research Lab of Lignocellulosic Functional Materials, Nanjing Forestry University, Nanjing 210037, China; jiaqi.guo@njfu.edu.cn; 3 Fushun Ecological Environment Monitoring Center of Liaoning Province, Fushun 113001, China; 0413zhangshuq@sina.com; 4 Center for Fluorescence Spectroscopy, Department of Biochemistry and Molecular Biology, University of Maryland School of Medicine, 721 West Lombard St., Baltimore, MD 21201, USA

**Keywords:** TLC-SERS, machine learning, pyrene, edible oil, quantitative

## Abstract

The presence of polycyclic aromatic hydrocarbons (PAHs) in edible oil has a serious effect on human health and may potentially induce cancer. This study combined thin-layer chromatography and surface-enhanced Raman spectroscopy (TLC-SERS) to rapidly and quantitatively detect PAHs in culinary oil. Machine learning using the principle component analysis-back propagation neural network (PCA-BP) was integrated with TLC-SERS for the detection of PAHs. Ag nanoparticles on diatomite (diatomite/Ag) TLC-SERS substrate were prepared via an in situ growth process and employed as a stationary phase in the TLC channel. The analyte sample was dropped onto the TLC channel for separation and detection. The diatomite/Ag TLC channel demonstrated excellent separation capability and superior SERS performance and successfully detected PAHs from edible oil at a sensitivity of 0.1 ppm. The PCA-BP quantitative analysis model demonstrated outstanding prediction performance. This work demonstrates that the combination of TLC-SERS technology with PCA-BP is an efficient and accurate method for quantitatively detecting PAHs in edible oil, which can effectively improve the quality of food.

## 1. Introduction

PAHs are a kind of persistent pollutant in the environment and food that are produced by the incomplete combustion of organic compounds, such as petroleum, wood, and organic polymers [1,2,3]. Some PAHs are carcinogenic and genotoxic and pose a serious threat to human health [4,5]. As early as 1979, the US Environmental Protection Agency (EPA) listed 16 harmful PAHs to the human body. PAHs are distributed in edible oils, and the consumption of edible oils with PAHs can have harmful effects on the human body [6]. At present, conventional methods for detecting PAHs include liquid chromatography-mass spectrometry (LC-MS), gas chromatography (GC), UV-Vis spectroscopy, and Fourier transform infrared (FT-IR) spectroscopy [7,8]. Nonetheless, these technologies exhibit limitations for on-site detection, including the bulkiness of the equipment and the intricate sample pretreatment process.

SERS is a powerful analytical technique owing to its exceptional selectivity and ability to obtain vibrational spectra of the compounds of interest at extremely low concentrations, even below nanomolar levels [9,10,11].

However, the complex component introduces interference, making it difficult to detect analytes from real samples using SERS. Therefore, it is necessary to isolate the target molecules before SERS detection. The pretreatment techniques for detecting PAHs in edible oils usually include liquid–liquid extraction, ultrasound, supercritical fluid extraction, microwave-assisted extraction (MAE), solid phase microextraction (SPME), gel permeation chromatography (GPC), and solid phase extraction (SPE) [12,13,14,15]. Interestingly, TLC is an effective and straightforward separation technique that distinguishes compounds based on their varying polarity [16]. The combination of TLC with SERS has been applied in the detection of target molecules from mixtures [17]. Hezel and co-workers first proposed TLC-SERS technology for monitoring analytes in herbs [18]. The TLC-SERS method has been widely used to detect mixed samples, as it has instant, simple, cost-effective, and accurate merits. Our group developed a TLC-SERS approach based on biosilica that has been effective for monitoring pesticides in food [19,20]. Conventional TLC plates are insufficient for SERS enhancement, as the stationary phase fails to amplify the Raman signal of the analyte [21].

In data processing, the Savitzky–Golay (SG) filter and Wavelet Transform are commonly used to reduce spectral noise and smooth spectral curves [22,23,24], while polynomial fitting and adaptive, weighted penalized least squares methods are employed to stabilize and unify spectral baselines [25,26]. However, these techniques rely on complex parameters and are only effective for single spectra with distinct features. PCA, a dimensionality reduction tool, projects data in multiple directions to identify principal components, effectively reducing dimensionality and highlighting the key components of substances within complex spectra [27]. As a backpropagation model, BPNNs (Backpropagation Neural Networks) can process data between individual points separately, avoiding the waste of computing resources while effectively capturing spectral features [28,29]. By combining the respective advantages of PCA and BPNN, the PCA-BPNN model has achieved good performance in fields, such as pattern recognition, content prediction, and financial analysis [30,31,32].

In this work, we employed a seed-mediated electroless deposition approach to apply Ag nanoparticles (NPs) onto the diatomite surface [33], subsequently utilizing these Ag NPs on diatomite as an innovative plasmonic thin-layer chromatography (PTLC) channel. The Ag NPs provide separation and a SERS enhancement effect simultaneously. The periodic pores enable diatomite with nanoscale photonic crystal features, which is beneficial for SERS enhancement. Furthermore, the porous structure of diatomite can improve mass transfer and analyte resolution during the TLC process. To enhance the detection accuracy, machine learning analysis was integrated with TLC-SERS for the separation and identification of PAHs in edible oil. A prediction model for PAHs was built using a principle component analysis-back propagation neural network (PCA-BP) based on a stoichiometric study.

PCA was employed to extract the primary features from the spectral data. The resulting key components were used as input layers for a BP neural network incorporating L2 regularization, which was then used to predict concentrations. Compared to the Partial Least Squares Regression (PLSR) method [34,35,36,37,38], the PCA-BP model demonstrated superior accuracy in quantitative analysis. This study integrates TLC-SERS and machine learning to quantify PAHs in edible oils. The combination of the PCA-BP model with TLC-SERS offers a rapid, reliable, and representative approach for on-site detection of PAHs in oil samples.

## 2. Experiment

### 2.1. Reagents and Instruments

Ascorbic acid (C_6_H_8_O_6_. AA), 4-Mercaptobenzoic acid (HSC_6_H_4_CO_2_H), pyrene, and hydrochloric acid (HCl) were supplied by Aladdin (Shanghai, China). Silver nitrate (AgNO_3_), Rhodamine6G (R6G), and carboxymethyl cellulose sodium salt (CMC-Na) were purchased from Innochem (Beijing, China). Diatomite earth (Celite209) was purchased from Sigma-Aldrich (St. Louis, MI, USA).

### 2.2. Preparation of Diatomite/Ag Composites

Diatomite (50 mg) was immersed in a mixed solution of SnCl_2_ and HCl (20 mM each, 1:1 ratio) for 5 min and then thoroughly rinsed with distilled water. Subsequently, silver (Ag) seeds were formed by treating the diatomite with AgNO_3_ (20 mM). Larger Ag NPs were obtained by soaking Ag-seeded filter paper in a mixture of AA and AgNO_3_. All reactions were conducted at room temperature.

### 2.3. Preparation of PTLC Channel

First, a band with a 2 mm width was prepared using adhesive tape on a 7.5 cm × 2.5 cm glass plate. Then, 10 mg diatomite/Ag was fully mixed with 1 mL aqueous solution of CMC (2%). The suspension was cast into the channel to prepare the PTLC plate.

### 2.4. TLC-SERS

A 1 µL liquid sample containing the analyte was dropped onto the PTLC channel. Then the PTLC channel was vertically placed in a developing beaker (130 mL). After separation, the PTLC channel was illuminated with UV light (365 nm) to trace the position of the target molecule. Then, 2 µL Au colloids were deposited onto the sample. The Raman spectra were collected on a Raman spectrometer (BWS465, B&W Tek, Newark, DE, USA) by using a 785 nm laser at a 5 cm^−1^ resolution. The laser power was 40 mW, and the acquisition time was 5 s.

### 2.5. Machine Learning and Analysis of Spectral Data

Peak fitting was performed on the characteristic peaks of the Raman spectrum using a Gauss function as the base algorithm to obtain key parameters, such as peak height, peak width, and peak area. PCA was then applied to optimize the processed data. The data were inputted into the PCA-BP model to obtain the final predicted concentration.

### 2.6. Instruments

The UV–vis spectra of the composites were measured by an Agilent UV-Vis-NIR spectrometers (Agilent, Palo Alto, CA, USA). The surface morphology of the samples was determined using a SU8010 scanning electron microscope (SEM) (Hitachi, Tokyo, Japan). Fourier transform infrared (FTIR) spectroscopy was performed using TMUX (Perkin-Elmer, Waltham, MA, USA).

## 3. Results and Discussion

### 3.1. SERS Properties of Diatomite/Ag

**Figure 1:**
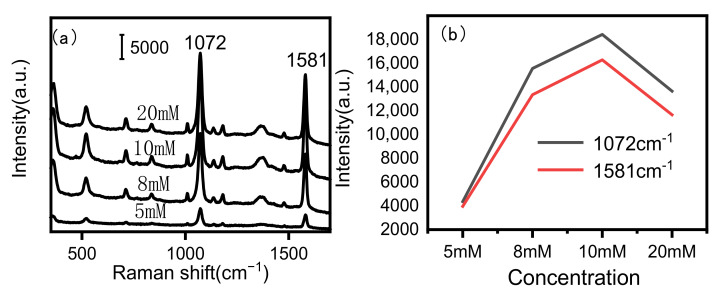
Raman spectra of 4-MBA from diatomite/Ag prepared with different concentrations of AgNO_3_ (5 mM, 8 mM, 10 mM, 20 mM) (**a**), and intensity of Raman peaks (1072 cm^−1^, 1581 cm^−1^) versus the concentration of 4-MBA (**b**).

The amount of AgNO_3_ used in the growth of Ag NPs also played an important role in the SERS performance of diatomite/Ag. 4-MBA was used as a probe molecule to examine the SERS effect of diatomite/Ag composites designed with various concentrations of AgNO_3_ and ascorbic acid (AA). Figure 1a illustrates the presence of distinct Raman peaks at 1072 cm^−1^ and 1581 cm^−1^, which are characteristic of 4-MBA [39]. The variation in the SERS signal versus the concentration of AgNO_3_ is shown in Figure 1a. As 5 mM AgNO_3_ was used in the reaction system, only a weak SERS signal was obtained. When increasing the concentration of AgNO_3_, the SERS signals gradually increased. The most intense SERS spectra were observed from the substrate with 10 mM AgNO_3_. Further increasing the concentration to 20 mM decreased the Raman signal (Figure 1b). Therefore, 10 mM of AgNO_3_ was selected for subsequent experiments.

**Figure 2:**
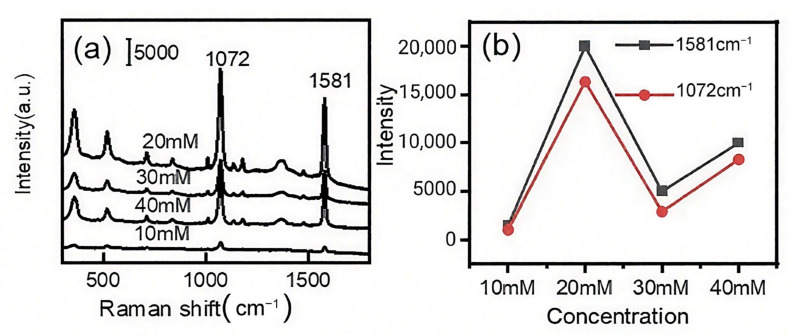
Raman spectra of 4-MBA measured from diatomite/Ag prepared under different conditions (AgNO_3_ 10 mM and varying AA concentration) (**a**), and intensity of Raman peaks (1072 cm^−1^, 1581 cm^−1^) versus the concentration of ascorbic acid (**b**).

Figure 2a illustrates the presence of distinct Raman peaks of 4-MBA. With a fixed AgNO_3_ concentration of 10 mM, the Raman signal intensity of 4-MBA increased when the concentration of AA increased. The strongest SERS signal occurred with 20 mM AA, probably due to the aggregation of densely packed Ag nanoparticles that create an increased number of “hot spots” [40]. However, additional increments in the AA concentration resulted in a decrease in the Raman signal (Figure 2b), which was attributable to the creation of larger Ag clusters. As the concentration of AA further increased, the intensity of the Raman signal was enhanced slightly. The reason was that excess AA could trigger new nucleation, generating secondary small particles, which were beneficial for SERS enhancement. Therefore, 20 mM AA was selected as the optimal concentration for subsequent experiments.

### 3.2. FTIR Spectra of the Substrate

**Figure 3:**
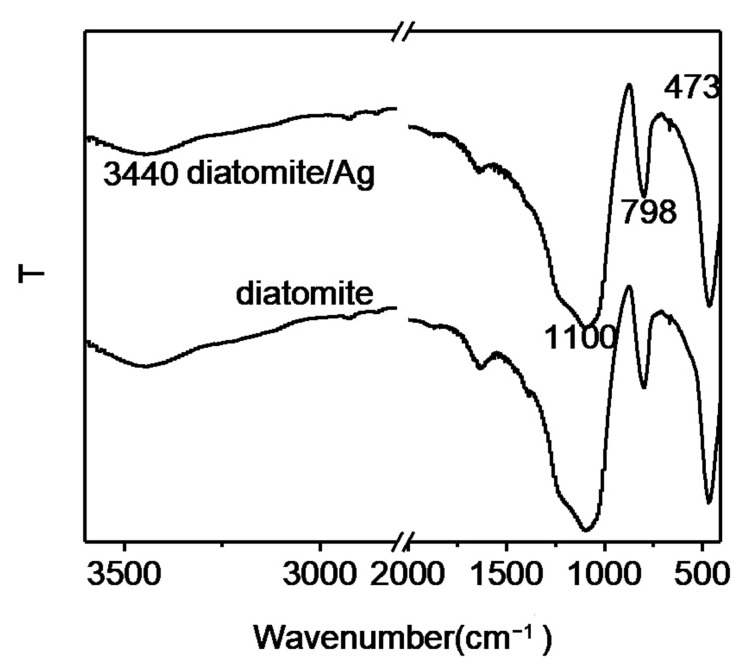
FTIR spectra of diatomite and diatomite/Ag.

The functional groups on the surface of the composite were studied by FTIR, as shown in Figure 3. Four strong absorption bands were observed from diatomite, the band at 3440 cm^−1^ was assigned to the vibration of the OH group, and the strong absorption bands at 1100 cm^−1^ and 798 cm^−1^ were due to the stretching vibration of the Si-O group. The sharp absorption band at 473 cm^−1^ was assigned to the O-Si-O antisymmetric bending vibration. The infrared spectrum of diatomite/Ag composite was similar to that of diatomite, indicating that the accumulation of Ag NPs did not change the properties of diatomite.

### 3.3. Morphology and Characterization of Diatomite/Ag NPs

**Figure 4:**
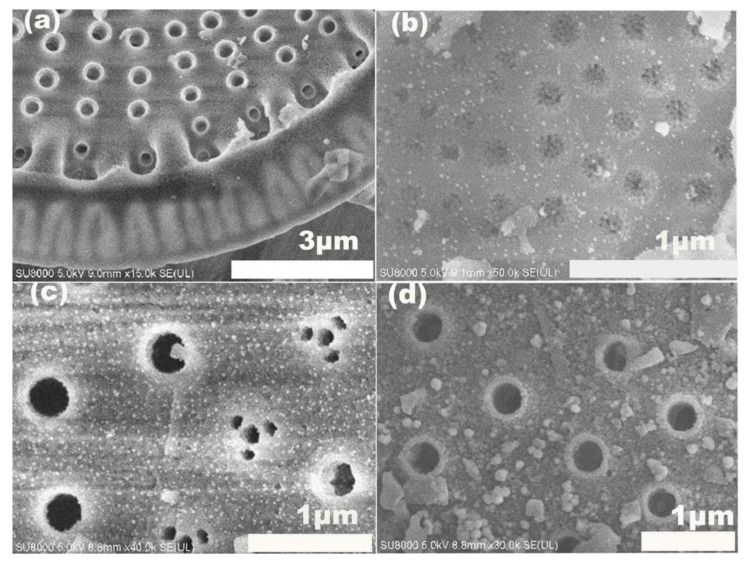
SEM images of diatomite (**a**) and diatomite/Ag prepared under various concentrations of AA (**b**) 10 mM, (**c**) 20 mM, and (**d**) 30 mM.

The surface morphologies and microstructures of diatomite and diatomite/Ag were determined by SEM images. As shown in Figure 4a, diatomite was disc-shaped, and pores with a diameter of 100–200 nm were periodically distributed on the diatomite skeleton. The periodic pores in diatomite provide photonic crystal characteristics, consequently increasing its sensitivity in SERS analysis and offering extensive SERS sites. During the process of Ag NP growth, the diameter and distribution of AgNPs on diatomite was controlled by adjusting the amount of AA. Three concentrations of AA (10 mM, 15 mM and 20 mM) were used in the reaction for the in situ synthesis Ag NPs on diatomite. The surface morphology of diatomite after decorating Ag NPs is shown in Figure 4b–d. A uniform distribution of Ag NPs on diatomite was observed. The bigger cluster was observed with a high concentration (30 mM) of ascorbic acid in the growth media.

### 3.4. Plasmonic Feature of the Substrate

**Figure 5:**
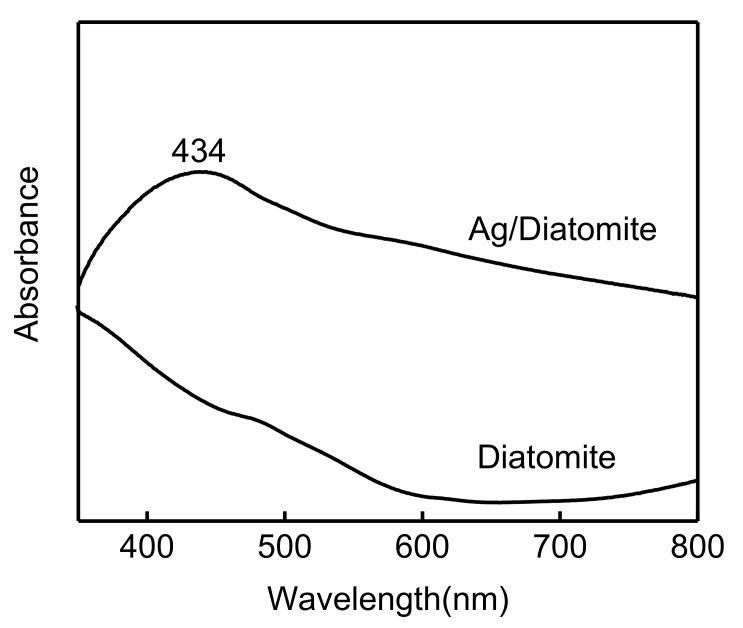
Uv–vis spectra of diatomite and diatomite/Ag composite.

UV–vis spectra were employed to investigate the optical properties of diatomite and diatomite/Ag. There were almost no peaks observed in diatomite (Figure 5), which was attributed to the main component of diatomite, silica. After depositing Ag NPs onto the surface of diatomite, an obvious peak was presented at 434 nm from diatomite/Ag, as shown in Figure 5. The adsorption peak was attributed to the local surface plasmon resonance (LSPR) of Ag on diatomite. It is well known that the wavelength of the maximum absorbance of plasmonic diatomite/Ag is related to the diameter, shape, and distribution of the Ag NPs. The peak position of LSPR of the Ag corresponded to a 20 mM AA concentration (Figure 1).

### 3.5. Uniformity of the Diatomite/Ag Channel

Uniformity is a critical factor in Raman measurement, and 4-MBA was selected as the probe molecule to evaluate the uniformity of SERS spectra from the diatomite/Ag TLC channel. Ten Raman spectra of MBA were collected from different points of the diatomite/Ag TLC channel, as shown in Figure S1a. The distribution of the intensity of the SERS peak at 1072 cm^−1^ is shown in Figure S1b. The relative standard deviation (RSD) calculated from ten peaks was 9.6%, indicating the excellent repeatability of the diatomite/Ag TLC channel.

The temporal stability of the diatomite/Ag TLC channel was evaluated and is shown in Figure S2. The SERS signals of MBA were measured from the diatomite/Ag TLC channel at different storage times. The histogram of the intensity distribution of the Raman peak at 1072 cm^−1^ was used to evaluate the temporal stability. The diatomite/Ag TLC channel still showed high sensitivity after 3 days.

### 3.6. Separating and Detecting Pyrene from Mixtures

**Figure 6:**
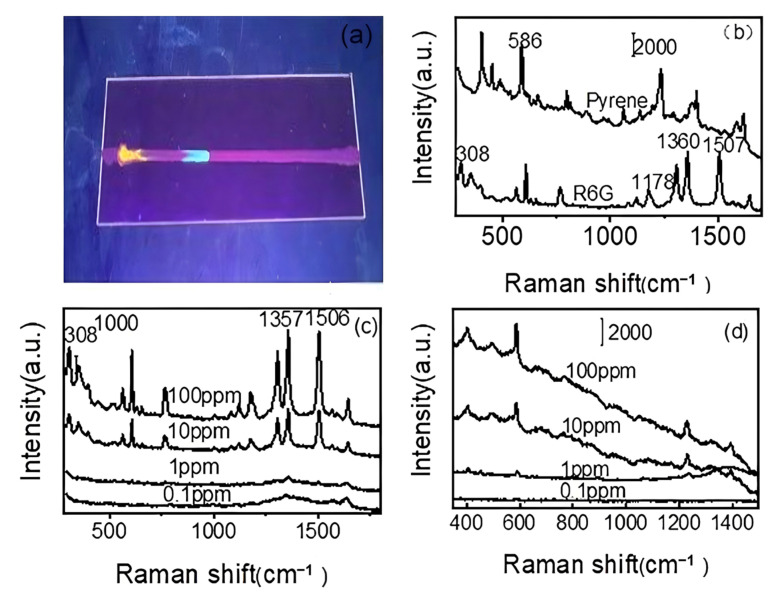
Photographic image of R6G and pyrene under UV light after TLC separation (**a**), Raman spectra of R6G and pyrene on the diatomite/Ag channel (**b**), Raman spectra of different concentrations of R6G (**c**) and pyrene (**d**) after separation by the diatomite/Ag TLC channel.

R6G was mixed with pyrene and used to evaluate the separation performance of the diatomite/Ag channel. The separation effect mainly relied on the materials on the TLC plate. Ag NP was deposited on the diatomite, which not only functioned as an enhanced substrate but also increased the separation efficiency in TLC, as Ag NPs have a smaller diameter than diatomite. The narrow width of the TLC channel could cause high confinement of the analyte due to the ultra-small dimension, which effectively increases the concentration of target molecules at the surface and improves the SERS signal. Ethyl acetate and n-hexane (*v*/*v* = 1:12) were used as eluents for the separation of pyrene from the mixture. After separation for 2 min, the diatomite/Ag TLC channel was placed under a UV lamp (365 nm). As shown in Figure 6a, the position of pyrene was far away from the original drop point due to its low molecular polarity. The development speed of pyrene was higher than in R6G, and the location of pyrene was farther than that of R6G, in which R6G was nearly located at the start line. The Au colloids were dropped onto the corresponding positions, and SERS spectra was recorded. As shown in Figure 6b, the intense Raman signals of pyrene and R6G were measured from the diatomite/Ag channel. The characteristic peak of pyrene was observed at 586 cm^−1^, which indicates that the diatomite/Ag TLC channel could successfully separate pyrene from the mixture. The characteristic peak of R6G was measured from the original point on the diatomite/Ag TLC channel, in which the band at 308 cm^−1^ was due to the C-O-C stretching vibration of R6G and the peaks at 1194, 1360, and 1508 cm^−1^ were assigned to the C-C stretching vibration of the aromatic ring of R6G. As shown in Figure 6c,d, the characteristic peak intensity of R6G and pyrene exhibited a monotonic decrease as the concentration decreased. There were still Raman signals observed as the concentration decreased to 0.1 ppm.

### 3.7. Identification of Pyrene from Edible Oil

**Figure 7:**
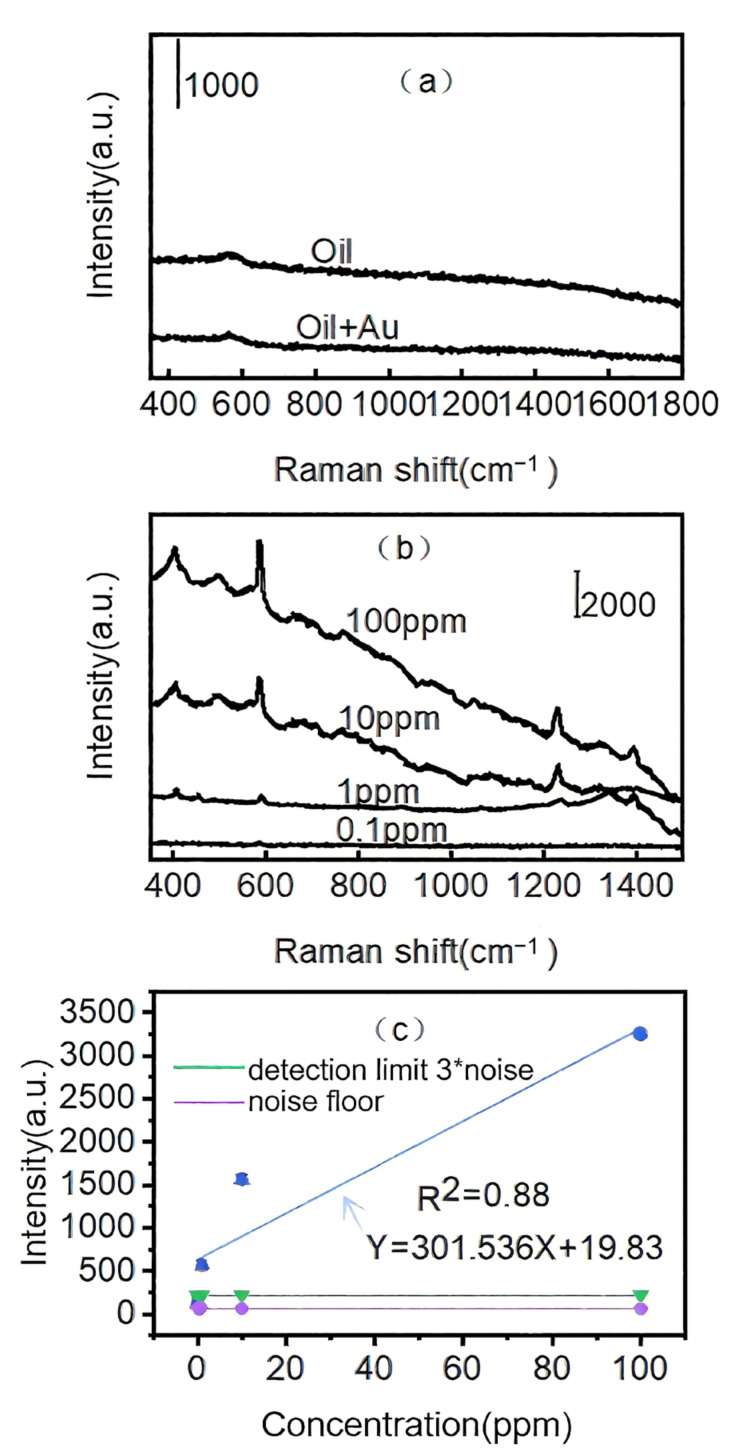
Raman spectra of the oil sample with pyrene (100 ppm) in the presence and absence of Au colloids (**a**), TLC-SERS detection of different concentrations of pyrene from the oil base on the diatomite/Ag channel (**b**), and linearity of the curve based on the intensities of SERS peaks at 586 cm^−1^; the limit of detection is indicated as a green line (**c**).

The plasmonic diatomite/Ag TLC channel was used to identify pyrene from edible oil. First, 1 mg pyrene was dissolved in 1 mL edible oil, and that was further diluted to different concentrations. The oil samples were deposited onto the diatomite/Ag TLC channel. The solvent of ethyl acetate and n-hexane (*v*/*v* 1:5) was mixed and used as the eluent. After separation through the TLC process, the spots of pyrene were traced under UV light. Then, 2 μL Au colloid was dropped onto the surface of the TLC channel, and the SERS spectra were measured and are presented in Figure 7b. The control experiment was developed on oil and oil/Au samples, as shown in Figure 7a. There were no Raman signals measured from oil samples with pyrene (100 ppm) containing the As Au colloid mixed with the oil sample. There were still no Raman signals obtained for pyrene. This was attributed to the phase separation, which occurred when the Au colloid encountered oil. The phase separation blocked the interaction between the analytes and plasmonic substrate, making it hard to measure the SERS signal from the oil sample. After developing the TLC process on the diatomite/Ag channel, pyrene was isolated from edible oil and the corresponding SERS spectra were obtained, as shown in Figure 7b. The characteristic peaks of pyrene were observed. When the concentration of pyrene reached 0.1 ppm, the characteristic Raman peak of pyrene could still be observed. Linear regression was performed to evaluate the linear relationship between the intensity of the SERS signal and the concentration of pyrene in oil (Figure 7c). The coefficient of determination (R^2^) was 0.88. The limit of detection, which was defined as the signal/noise (SNR) ratio at 3 [41] and marked with a green line in Figure 7c, the calculated limit of detection was 0.08 ppm.

### 3.8. Establishment of the PCA-BP Model

**Figure 8:**
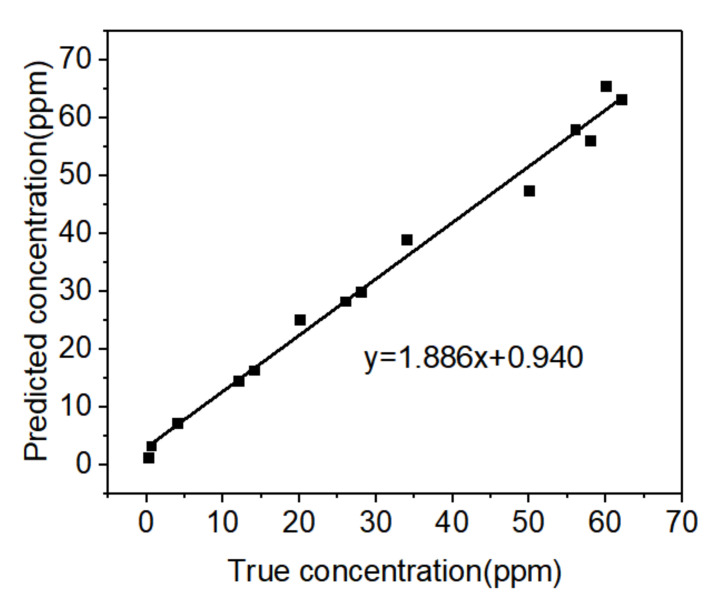
Relationship between real and predicted concentrations of pyrene.

The quantitative analysis of pyrene from SERS spectra has attracted great interest. However, the trace amount of pyrene in mixed samples and the separation and detection process in TLC-SERS affected the linear relationship between the Raman spectra and the concentration of pyrene, which hinders quantitative detection. To solve this problem, PCA-BP mode was employed to process the complex spectra data of pyrene. PCA-BP was first established as a model for predicting the unknown concentration of pyrene from Raman spectra. Different concentrations (0.01 ppm to 70 ppm) of pyrene in edible oil were prepared, and the Raman spectra of pyrene were measured after the TLC process. A total of 20 measurements were performed for each concentrations. For the acquired spectral data, we divided it into four regions based on characteristic peaks: 350–405, 410–490, 500–600, and 1190–1250. Each region was separately processed using PCA for dimensional reduction and fitting. The results showed that the spectral range of 500–600 exhibited the highest variance contribution rate. Subsequently, the spectral data within the 500–600 range was used as input for a PCA-BPNN network. The hidden layers of the BP network consisted of Conv1D layers configured as (32, 4), (64, 4), (128, 2), and (256, 1), followed by a flattened layer. Fully connected layers with 400, 200, 100, and 50 neurons were then applied, and the output layer used a sigmoid activation function with linear scaling to achieve the prediction results. As shown in Figure 8, the square dots predict values, and the solid line corresponds to real concentrations. The PCA-BP equation was y = 1.886x + 0.940. The test set decision system correlation coefficient (R^2^) was used as an indicator to evaluate model performance. When R^2^ is 0.995, it indicates that the predicted value is very approximate to the real concentration. The PLSR model was also employed to analyze the data for comparison purposes. As shown in Figure S3, the value of R^2^ was 0.88. Therefore, the PCA-BP model realized quantitative analysis to detect pyrene using the TLC-SERS.

## 4. Conclusions

In this work, we developed a convenient and successful approach for the quantitative detection of pyrene in oil. Diatomite was initially functionalized with Ag NPs via an in situ growing method, utilizing the plasmonic characteristics of Ag NPs to amplify the SERS signal. Ag NPs were produced by utilizing 10 mM AgNO_3_ and 20 mM AA. The diatomite/Ag composite was employed to construct a TLC plate featuring narrow channels, which is efficient at separating pyrene from mixtures in 2 min. The TLC-SERS technique, utilizing a plasmonic diatomite/Ag plate, was combined with PCA and BP (PCA-BP) for the rapid qualitative and quantitative detection of pyrene in oil. This technique exhibited a detection limit as low as 0.08 ppm for pyrene in complex mixtures. This approach’s simplicity and efficiency make it highly appropriate for the on-site detection of PAHs across diverse environmental and industrial settings.

## Data Availability

Data are contained within the article.

## Supplementary Materials

The following supporting information can be downloaded at: https://www.mdpi.com/article/10.3390/bios15080477/s1. Figure S1: 4-MBA Raman spectra at 10 different positions of diatomite/Ag TLC channel (a) and Raman intensity histograms (b), Figure S2: 4-MBA Raman spectra at 10 different positions of diatomite/Ag TLC channel (a) and Raman intensity histograms (b), Figure S3: Relationship between real and predicted concentrations of pyrene analysis by PLSR model.
