# Supplementary material for: Quantitative Detection of Pyrene in Edible Oil via Plasmonic TLC-SERS Combined with Machine Learning Analysis
Source: Biosensors (Basel). 2025 Jul 23;15(8):477. doi: 10.3390/bios15080477 (PMC12384367; doi:10.3390/bios15080477)
Supplement: Supplementary file 1 [file biosensors-15-00477-s001.zip › biosensors-3717222-supplementary.pdf]

Quantitative Detection of Pyrene in Edible Oil via Plasmonic TLC-SERS Combined with Machine Learning Analysis

Jiahui Tian [1], Xianhe Jiao [1], Jiaqi Guo [2], Qian Yu [1], Shuqin Zhang [3], Guizhou Gu [1,*], Kundan Sivashanmugan [4,*] and Xianming Kong [1]

[1] School of Petrochemical Engineering, Liaoning Petrochemical University, Fushun 113001, China; tianjiahui@stu.lnpu.edu.cn (J.T.); lnpulnpu0413@163.com (X.J.); qyu@lnpu.edu.cn (Q.Y.); xmkong@lnpu.edu.cn (X.K.)

[2] Jiangsu Co-Innovation Center for Efficient Processing, Utilization of Forest Resources and Joint International Research Lab of Lignocellulosic Functional Materials, Nanjing Forestry University, Nanjing 210037, China; jiaqi.guo@njfu.edu.cn

[3] Fushun Ecological Environment Monitoring Center of Liaoning Province, Fushun 113001, China; 0413zhangshuq@sina.com

[4] Center for Fluorescence Spectroscopy, Department of Biochemistry and Molecular Biology, University of Maryland School of Medicine, 721 West Lombard St., Baltimore, MD 21201, USA

* Correspondence: guguizhou2013@163.com (G.G.); skundan@som.umaryland.edu (K.S.)

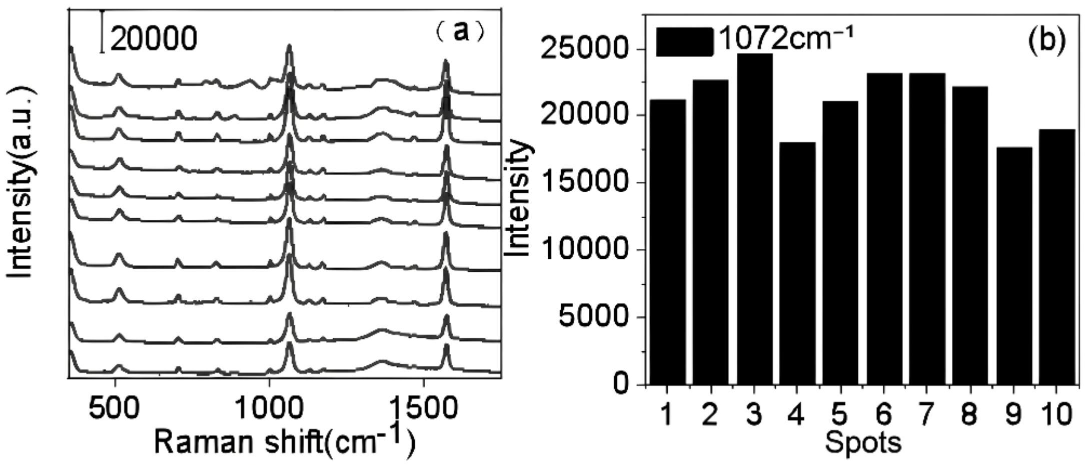

Figure S1. 4-MBA Raman spectra at 10 different positions of diatomite/Ag TLC channel (a) and Raman intensity histograms (b)

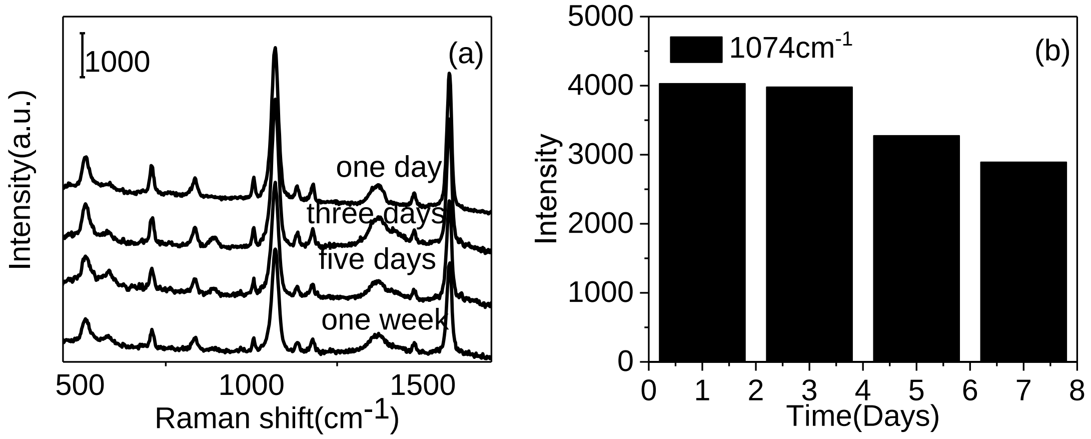

Figure S2. 4-MBA Raman spectra at 10 different positions of diatomite/Ag TLC channel (a) and Raman intensity histograms (b)

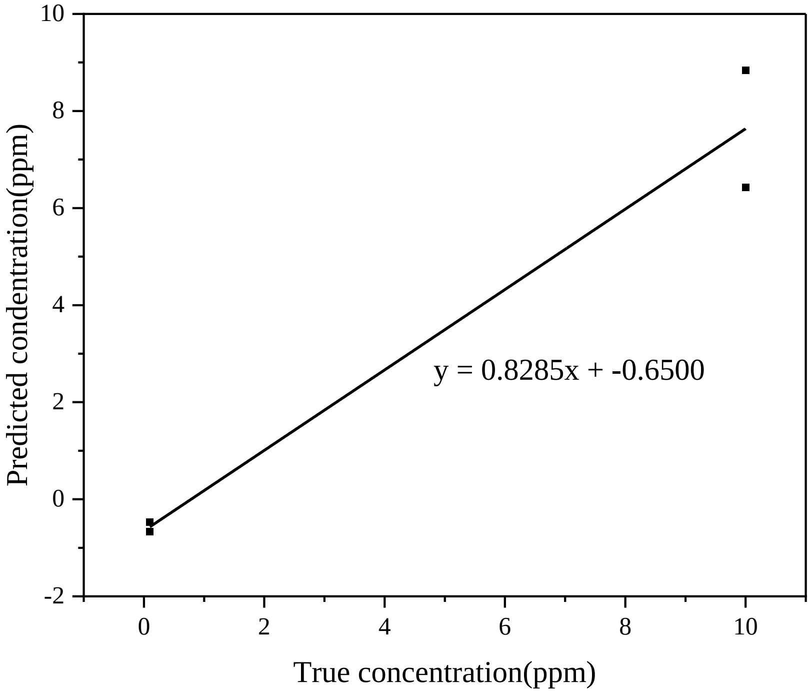

Figure S3. Relationship between real and predicted concentrations of pyrene analysis by PLSR model.
